# Electrical properties and thermal stability in stack structure of HfO_2_/Al_2_O_3_/InSb by atomic layer deposition

**DOI:** 10.1038/s41598-017-09623-1

**Published:** 2017-09-12

**Authors:** Min Baik, Hang-Kyu Kang, Yu-Seon Kang, Kwang-Sik Jeong, Youngseo An, Seongheum Choi, Hyoungsub Kim, Jin-Dong Song, Mann-Ho Cho

**Affiliations:** 1 Institute of Physics and Applied Physics, Yonsei University, Seoul, 120-749 Republic of Korea; 2 School of Advanced Materials Science and Engineering, Sungkyunkwan University, Suwon, 440-746 Republic of Korea; 3 Center of Opto-electronic Materials, Korea Institute of Science and Technology, Seoul, 02792 Republic of Korea

## Abstract

Changes in the electrical properties and thermal stability of HfO_2_ grown on Al_2_O_3_-passivated InSb by atomic layer deposition (ALD) were investigated. The deposited HfO_2_ on InSb at a temperature of 200 °C was in an amorphous phase with low interfacial defect states. During post-deposition annealing (PDA) at 400 °C, In–Sb bonding was dissociated and diffusion through HfO_2_ occurred. The diffusion of indium atoms from the InSb substrate into the HfO_2_ increased during PDA at 400 °C. Most of the diffused atoms reacted with oxygen in the overall HfO_2_ layer, which degraded the capacitance equivalent thickness (CET). However, since a 1-nm-thick Al_2_O_3_ passivation layer on the InSb substrate effectively reduced the diffusion of indium atoms, we could significantly improve the thermal stability of the capacitor. In addition, we could dramatically reduce the gate leakage current by the Al_2_O_3_ passivation layer. Even if the border traps measured by *C*–*V* data were slightly larger than those of the as-grown sample without the passivation layer, the interface trap density was reduced by the Al_2_O_3_ passivation layer. As a result, the passivation layer effectively improved the thermal stability of the capacitor and reduced the interface trap density, compared with the sample without the passivation layer.

## Introduction

SiO_2_/Si-based metal oxide semiconductor (MOS) devices have been aggressively scaled down in the semiconductor industry. Now, gate dielectric films with sub 1 nm capacitance equivalent thickness (CET) and channel substrates with high mobility and low power consumption are required for MOS device applications. These requirements have led to the employment of III–V channel materials and high-κ gate dielectrics^1^. For next-generation large-scale integrations, Hf-based gate (high-κ) dielectrics on III–V compound semiconductors such as InGaAs, GaSb, InP, InAs, and InSb are being seriously considered^2–6^. InSb has the highest bulk mobility (77000 cm^2^ V^−1^ s^−1^) among the III-V materials, so this material is considered to be a particularly attractive III–V compound for high-speed metal-oxide semiconductor field-effect transistors (MOSFETs). Despite InSb’s advantages, there are few reports on it, compared with other materials such as InP, InGaAs, and InAs, because its low melting point and narrow bandgap can act as weak points in device application. The melting point of InSb, ∼527 °C at atmospheric pressure, is insufficient for obtaining the process condition for MOSFET integration, and the bandgap of InSb, ∼0.17 eV at 293 K, is not enough to block thermal effects^7^. Moreover, these weak characteristics can result in defect states being easily generated in the MOSFET device. The generation of some defect states in the bandgap of InSb easily induces electrical problems such as a pinning effect, compared to what occurs in other compound semiconductors within a relatively wide bandgap. Therefore, clarifying the role of defects within the band structure of HfO_2_/InSb on electrical properties in the HfO_2_/InSb system is crucial, because the defects can directly affect capacitor modulation and the leakage current level. In addition, it is very important to control the defects by improving thermal stability.

In this study, to improve the thermal stability and electrical properties in the HfO_2_/InSb system, we introduced an interfacial passivation layer using Al_2_O_3_. Even though the Al_2_O_3_ passivation has been used with some III-V channel materials, the effect of the Al_2_O_3_ passivation shows various results depending on the chemical reaction with substrate materials. In particular, because the passivation effect has not yet been clarified in InSb system, we focused on the Al_2_O_3_ passivation effect in the InSb system. We examined the interfacial reaction causing interfacial traps in the HfO_2_/InSb system by analyzing the differences in elemental In diffusion and the chemical state at the interface between HfO_2_/InSb and HfO_2_/Al_2_O_3_/InSb. A MOS capacitor with the passivation layer of Al_2_O_3_ shows improved interface properties related to leakage current and maximum capacitance, compared with a capacitor without a passivation layer. In addition, capacitance equivalent thickness and low parallel conductance level are maintained in HfO_2_/Al_2_O_3_/InSb after annealing at 400 °C. Also, the MOS capacitor with the passivation layer shows significantly reduced defect density.

## Method

### Sample preparation and measurements

We prepared two types of high-κ oxide films on InSb substrate: (i) HfO_2_/InSb and (ii) HfO_2_/Al_2_O_3_/InSb (HA/InSb). Before the deposition of high-κ oxide films, the native oxides on n-type InSb(100) substrates were removed by wet cleaning using a dilute solution of buffered oxide etchant (BOE, NH_4_F:HF = 6:1) to 1% for 2 min. After rinsing with deionized water, the samples were immediately transferred to the atomic layer deposition (ALD) chamber. It took less than 10 s to transfer the sample to the ALD load lock chamber. HfO_2_ and Al_2_O_3_ films were grown on the BOE-treated InSb surface using the ALD system with tetrakis(ethylmethylamino) hafnium (TEMAHf) as the Hf metal precursor and trimethyl aluminum (TMA) as the Al metal precursor. We used H_2_O for the oxidant and N_2_ gas was the purge gas for the film growth. The substrate was maintained at 200 °C and 1 Torr was used for the working pressure of the deposition process. For HfO_2_/InSb, we performed 75 cycles of ALD to deposit the HfO_2_ layer. For HA/InSb, we performed 63 and 10 cycles to deposit HfO_2_ and Al_2_O_3_ layers, respectively. After the deposition, some films were annealed at 400 °C by using a rapid thermal process (RTP) for 1 min in an N_2_ environment. We used high-resolution transmission electron microscopy (HR-TEM) (Tecnai F20) to analyze the micro-structure, morphology, and film thickness of the HfO_2_ and HfO_2_/Al_2_O_3_ films on InSb with a 200 kV accelerating voltage at the Korea Institute of Science and Technology (KIST). The quantities of elements were examined by time-of-flight secondary ion mass spectroscopy (TOF-SIMS). Bi^1+^ was used for the analysis beam, and the current used during analysis was 1 pA; the analysis area was 100 × 100 μm and the analysis time was 60 s. To revise the charging effect, a flood gun was used to provide a steady flow of low-energy electrons to the desired target. The chemical states for the films grown on InSb were examined by x-ray photoelectron spectroscopy (XPS). XPS core-level spectra of In 3d, Sb 3d (O 1 s), Al 2p, and C 1 s were obtained by using a monochromatic Al Kα x-ray source (*h*ν = 1486.7 eV) with a 20 eV path energy. The InSb substrates were electrically grounded to the electron analyzer to calibrate the charging effects. Binding energies were calibrated by core-level spectra using the C 1 s spectrum (284.5 eV). To analyze the XPS core-level spectra, the background was removed by using a Shirley-type procedure. Full widths half-maximum (FWHM) of the constituent peaks were kept constant. Fitting curves were determined by Gaussian and Lorentzian distributions, in which the Gaussian distribution ratio was >60%. In the case of In 3d and Sb 3d, the intensity ratio of the spin-orbit splitting was determined by the probability of transition to such a state. Energy separation for In 3d was fixed at 7.54 eV. For electrical characterization, a metal oxide semiconductor capacitor (MOSCAP) was fabricated by sputtering of a 120-nm-thick metal (TiN) top contact of various metal area sizes through a shadow mask. Capacitance*–*voltage (*C–V*) characteristics and the conductance were measured using an Agilent E4980A. To obtain the CET and dielectric constant of HfO_2_ and HfO_2_/Al_2_O_3_ film, we calculated the CET of the HfO_2_ film using the following equation:

1 $${C}_{{\rm{high}}{\rm{\kappa }}}=\frac{{\varepsilon }_{0}{\kappa }_{{\rm{high}}{\rm{\kappa }}}A}{{d}_{{\rm{high}}{\rm{\kappa }}}}=\,\frac{{\varepsilon }_{0}{\kappa }_{{\rm{SiO}}2}A}{{d}_{{\rm{SiO}}2}}\to {\rm{CET}}=\frac{3.9{\varepsilon }_{0}}{{C}_{{\rm{high}}{\rm{\kappa }}}A}$$

where *C*_high k_ is the capacitance obtained from *C–V* measurement, A is the gate metal size, κ is relative permittivity, d is the oxide thickness, and ε_0_ = 8.85 × 10^−12^ F/m is the vacuum permittivity. The interface trap density (*D*_it_) was determined by parallel conductance (*G*_p_/ω)_max_, and the energy level of the defect state was determined from frequency measurements. The *G*_p_/ω value was calculated using the equation

2 $$\frac{{G}_{{\rm{p}}}}{{\rm{\omega }}}=\frac{{\rm{\omega }}{C}_{{\rm{ox}}}^{2}{G}_{{\rm{c}}}}{[{G}_{{\rm{c}}}^{2}+{{\rm{\omega }}}^{2}{({C}_{{\rm{ox}}}-{\rm{C}})}^{2}]}$$

where ω is 2π*f*, and frequency is measured from 10 kHz to 1 MHz. *C*_ox_ is the gate oxide capacitance, and *G*_C_ and *C* are calibration data, which are related to *G*_m_ and *C*_m_ (which are the measured conductance and capacitance, respectively). A correction term was considered for the high leakage current caused by the thin film. *D*_it_ in depletion is proportional to the peak values of *G*_p_/ω,

3 $${D}_{{\rm{it}}}=2.5\frac{{({G}_{{\rm{p}}}/{\rm{\omega }})}_{{\rm{\max }}}}{Aq}$$

where *A* is the area of the electrode and *q* is the elemental charge. The trap energy level is given by Shockley–Read–Hall statistics for the capture and emission rates using the following equation, which describes the relationship between the time constant τ of the trap and the frequency^8^:

4 $$f=\frac{1}{2\pi \tau }=\frac{{v}_{{\rm{th}}}\sigma N}{2\pi }\exp \,[\frac{-{\rm{\Delta }}E}{{k}_{{\rm{B}}}T}]$$

where *v*_th_ is the average thermal velocity of the majority carrier, *N* is the effective density of states of the majority carrier band, σ is the captured cross section of the trap state, and T is sample temperature. We evaluated the energy level of the defect states within *D*_it_ by using the relationship between the trap time constant τ and the frequency^9^. ﻿The stress-induced leakage current characteristics of HfO_2_/InSb and HA/InSb were investigated to evaluate electrical reliability under voltage stress. Forward and reverse I–V were measured as a function of the voltage.

## Calculation

Density functional theory (DFT) calculations were employed to evaluate the energy levels and the energy of formation of the defect states. Calculations were performed using VASP code with the exchange correlation function of the generalized gradient approximation (GGA) PBESol. Geometry optimization for the unit cell of the P121/C1 HfO_2_ structure and of the alpha Al_2_O_3_ was performed. The unit cells of HfO_2_ and Al_2_O_3_ were calculated as 5 × 5 × 5 and 7 × 7 × 7, respectively. To minimize interactions between charged defects, 2 2 2 (HfO_2_) and 2 2 1 (Al_2_O_3_) supercells were used for the defect calculation. Gamma k-points for geometry optimization and a 3 × 3 × 3 k-point were used for calculations of the energy state and the density of the states.

## Results and Discussion

**Figure 1:**
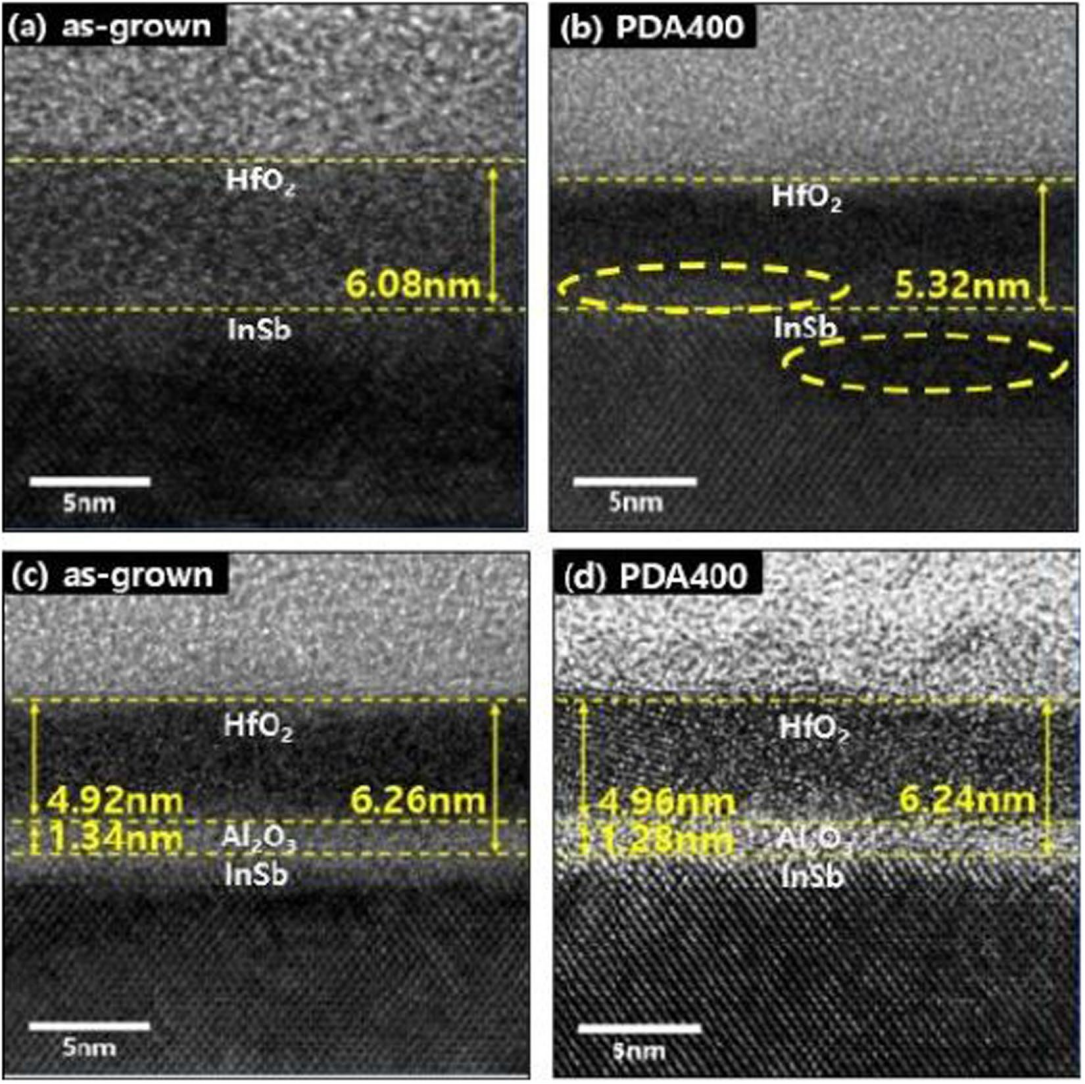
Cross-sectional TEM images of as-grown (**a**) HfO_2_/InSb and (**c**) HfO_2_/Al_2_O_3_/InSb and (**b**) HfO_2_/InSb and (**d**) HfO_2_/Al_2_O_3_/InSb after annealing at 400 °C.

To investigate the structural change caused by interfacial reaction, cross-sectional HR-TEM images of HfO_2_ film on InSb were observed, as shown in Fig. 1. The thickness of the HfO_2_ film on InSb without an Al_2_O_3_ layer was ∼6.0 nm at room temperature. After post-deposition annealing at 400 °C, the thickness decreased and the HfO_2_ film became locally crystallized. In particular, disordered crystalline structure was locally observed, as a result of damage during the annealing process. The damage could be caused by the dissociation of InSb during the annealing process owing to the poor thermal stability of InSb. In the HfO_2_/Al_2_O_3_ stack structure, the thicknesses of HfO_2_ and Al_2_O_3_ films on InSb were ∼5.0 and ∼1.2 nm at room temperature, respectively, as shown in Fig. 1c. After the annealing process, crystallized structure was observed in local regions of the film. It is noted that, even after PDA at 400 °C, the thicknesses were maintained and no changes in stacking structure were observed. These distinct differences in the structure between the stacking film and a single film indicate that the Al_2_O_3_ layer can effectively act as a passivation layer.

**Figure 2:**
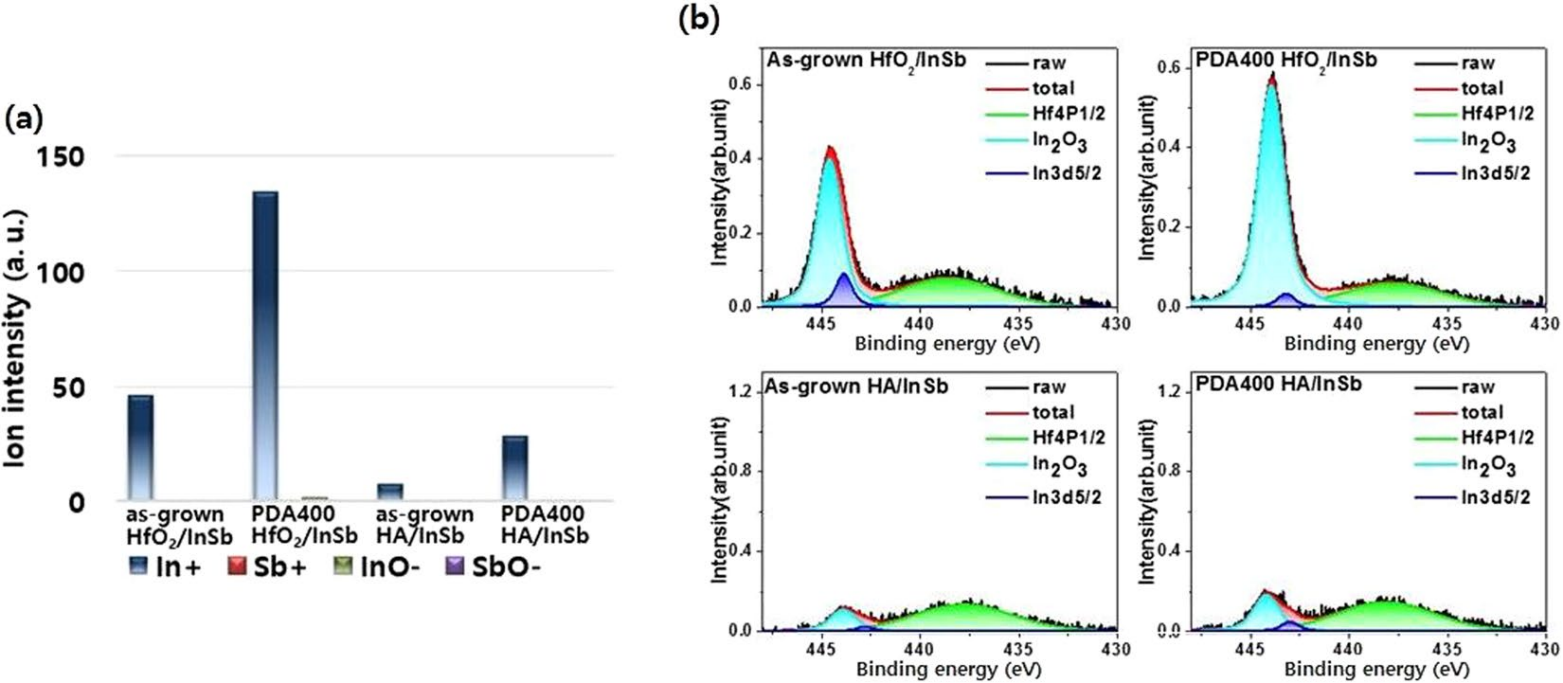
(**a**) TOF-SIMS data for the ratio of elements on the surface of the oxide layer and (**b**) XPS In 3d core-level spectra with a takeoff angle of 15°.

To investigate the dissociation of InSb and the effect of the Al_2_O_3_ passivation layer in detail, we analyzed the ratio of elements on the oxide layer surface and the chemical states by using TOF-SIMS and XPS, respectively, as shown in Fig. 2. The data show the differences in the quantities of In+, Sb+, InO−, and SbO− ions on the surface between the two samples of HfO_2_/InSb and HA/InSb, (see Supporting Table S1). In both samples, the intensities of Sb+, InO−, and SbO− were very low on the surface of the oxide layer, while that of In+ on the surface of the oxide layer was much higher. This result implies that the In+ ion can be more easily out-diffused through the oxide films than can other ions. The quantity of In+ in the as-grown HfO_2_/InSb sample was greater than that in as-grown HA/InSb, as shown in Fig. 2a, indicating that diffusion of In is greater in HfO_2_/InSb during the ALD growth process, compared to the case of as-grown HA/InSb. After PDA at 400 °C, the intensity of In+ significantly increased in HfO_2_/InSb, indicating that the increasing In+ quantity on the oxide surface resulted from the thermal process in the HfO_2_/InSb sample. In contrast, the quantity of In+ in HA/InSb was very low, compared with that in HfO_2_/InSb, implying that diffusion was effectively blocked. Moreover, after PDA at 400 °C, the increase of In+ in HA/InSb was relatively suppressed, compared with that in HfO_2_/InSb, implying that the diffusion of In+ in HA/InSb was still blocked even during PDA at 400 °C. As a result, we can confirm that the quantity of surface In+ ions is effectively controlled by the Al_2_O_3_ passivation layer in TOF-SIMS data.

Moreover, we analyzed the chemical states of the diffused indium by using XPS measurements. Figure 2b for the In 3d core-level spectra show various peaks: 444.6 eV for In_2_O_3_, 444.0 eV for the InSb substrate, and 439.0 eV for the HfO_2_ film. The XPS data also well represent the behavior of In diffusion. Using the 15° tilted XPS data, we can extract the chemical state within a depth of 1–2 nm, revealing that the In_2_O_3_ bonding was localized on the surface. Comparing In 3d data of HfO_2_/InSb with those of HA/InSb, we confirm that the In_2_O_3_ state on oxide surface is much higher in HfO_2_/InSb than in HA/InSb. Thus, elemental indium passing through oxide layer is oxidized on the surface region in HfO_2_/InSb, whereas the Al_2_O_3_ passivation layer effectively suppresses this process. The effective role of the Al_2_O_3_ passivation layer is related to the structure of Al_2_O_3_ and chemical reactivity. It is easier for elemental indium to interstitially diffuse out through the HfO_2_ layer than through the Al_2_O_3_ layer, because the lattice constant of HfO_2_ is larger than that of Al_2_O_3_. Additionally, using the 60° tilted XPS data, we extracted the chemical state in a region (5–6 nm) below the surface region (see Supporting Fig. S1). The data at two different tilted angles show that the oxidized In is observed on the surface as well as in the oxide layer in HfO_2_/InSb (see Supporting Fig. S1). In particular, the peak for In_2_O_3_ in HA/InSb is very low both at the surface and inside the oxide layer, compared with that in HfO_2_/InSb, which is consistent with the TOF-SIMS data. The dependence of the peak intensity on the tilted angle also shows a distinct difference: i.e., the formation of In_2_O_3_ in HfO_2_/InSb is uniformly distributed, whereas that in HA/InSb is more localized at the interface region. The difference in oxidation state of diffused In between the two samples can suggest another reaction process for In_2_O_3_ related to the reactivity. Comparing the Gibbs free energy of HfO_2_ (−1088.2 KJ/mol), Al_2_O_3_ (−1582.3 KJ/mol), and In_2_O_3_ (−830.7 KJ/mol), we conclude that the reaction of In with Al_2_O_3_ or HfO_2_ is not possible^6^. Therefore, the oxygen for the formation of In_2_O_3_ can be externally supplied from the oxide surface. The diffusion of oxygen through the oxide layers of HfO_2_ and Al_2_O_3_ being easier than that of indium explains the difference in the distributed position of the of In_2_O_3_ formation between the two samples. According to the reported data for high κ on various III–V compound semiconductors such as GaAs, InAs, and GaSb, the elements of the III–V compound semiconductors are also easily diffused out through the hafnium oxide layer during the annealing process. In the case of the GaAs substrate, large amounts of Ga–O and As–O states were generated during annealing at 700 °C^10^. In the case of the InAs substrate, As_2_O_3_ and As_2_O_5_ states were rarely detected in the as-grown HfO_2_/InAs, whereas elemental As and In_2_O_3_ states were clearly measured by the result of interfacial reactions between interdiffused oxygen and the InAs substrate. During the post-deposition annealing process at 600 °C, oxidation states of As_2_O_3_, As_2_O_5_, and In_2_O_3_ were generated at the surface region of the HfO_2_^6^. In the case of GaSb, the Ga–O and Ga_2_O_3_ states were generated on the GaSb surface during the ALD process even at 250 °C^11^. As a result, these reported cases mean that elemental In, Ga, and As are easily diffused out through the hafnium oxide layer during the annealing process. Finally, we can conclude that the HfO_2_ layer is not effective for preventing elemental indium from diffusion, whereas the Al_2_O_3_ layer is very effective for blocking the diffusion of In. In addition, although the diffusion of oxygen cannot be controlled by the Al_2_O_3_ layer, the thermal stability of the dielectric layer/InSb can be enhanced in the HA/InSb structure by preventing the dissociation of InSb.

**Figure 3:**
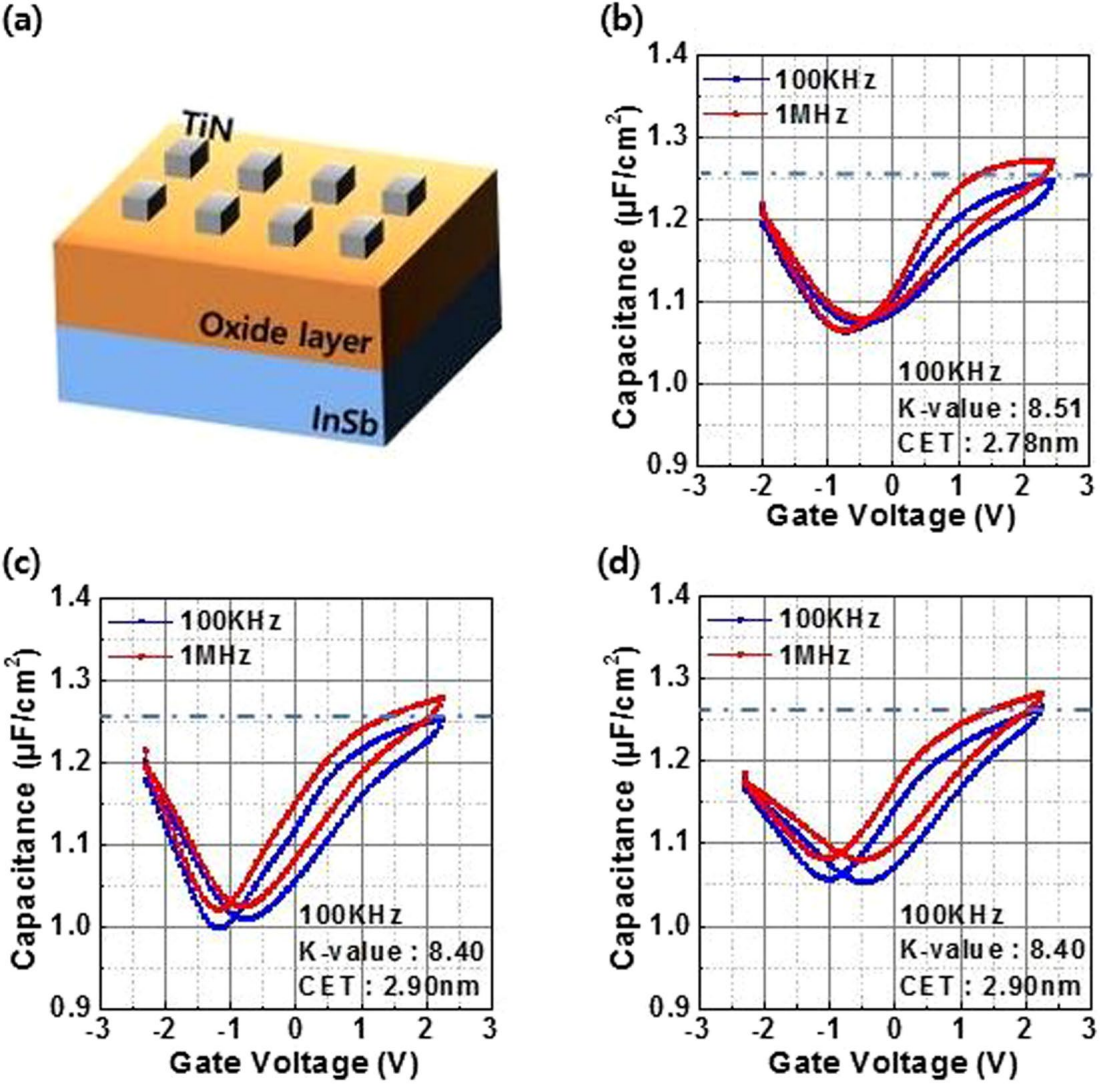
(**a**) Schematic of MOS capacitor and 100 kHz and 1 MHz *C*–*V* characteristics of (**b**) as-grown HfO_2_/InSb, (**c**) as-grown HfO_2_/Al_2_O_3_/InSb, and (**d**) post-deposition annealed HfO_2_/Al_2_O_3_/InSb at 400 °C for the forward scan (from inversion to accumulation direction) and the reverse scan (from accumulation to inversion direction).

To investigate the effect of chemical reactions on the diffused In in dielectric characteristics between HfO_2_/InSb and HA/InSb, frequency-dependent *C*–*V* curves were evaluated in the ~6.0-nm-thick HfO_2_/InSb and HA/InSb before and after PDA at 400 °C, as shown in Fig. 3. In previous experiments, the reported dielectric constants of HfO_2_ and Al_2_O_3_ were found to be ∼22 and ∼12, respectively^10, 12^. According to the effect of series capacitance, the single-layer HfO_2_ film has a higher capacitance value than the stack structure of HfO_2_/Al_2_O_3_. However, measured accumulation capacitance data at a frequency of 100 kHz ac in single-layer HfO_2_ is similar to the that in the stack structure of HfO_2_/Al_2_O_3_: i.e., the calculated dielectric constants of HfO_2_ and HfO_2_/Al_2_O_3_ are also almost the same as 8.51 and 8.40, respectively. Calculated CETs using the dielectric constants of HfO_2_ and HfO_2_/Al_2_O_3_ are 2.78 and 2.9 nm, respectively. In particular, the oxide layer of HA/InSb maintained the same dielectric constant and CET after PDA at 400 °C, as shown in Fig. 3c and d. Unfortunately, we could not obtain reliable data in the single-layer HfO_2_ after PDA at 400 °C because the conductance value of the sample was too high to enable measurement of the capacitance. The result clearly suggests that significant deterioration of the film quality occurred during PDA in the single-layer HfO_2_, not in the stack structure of the HA/InSb sample. Moreover, the result implies that elemental In diffusing through HfO_2_ can deteriorate the oxide film by generating defect states, because the behavior of charges through the defects increases the leakage path, resulting in a decrease in the reliability of the film. However, since the Al_2_O_3_ layer prevented elemental In from out-diffusion in the HfO_2_/Al_2_O_3_ stack structure, the dielectric constant of HA/InSb is maintained even after PDA at 400 °C.

**Figure 4:**
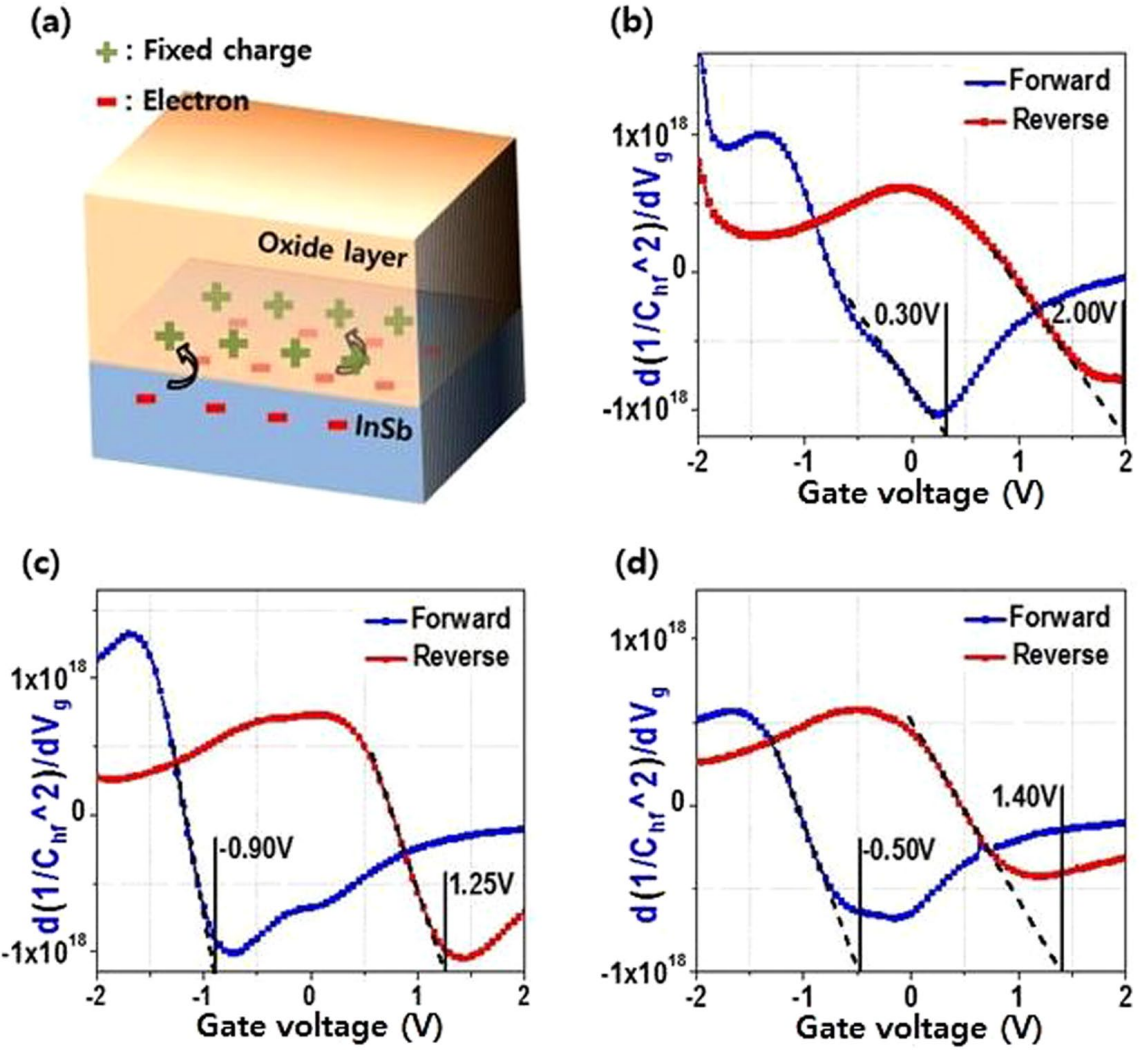
(**a**) Schematic of fixed charge and trap charge and flat band voltage of (**b**) as-grown HfO_2_/InSb, (**c**) as-grown HfO_2_/Al_2_O_3_/InSb, and (**d**) post-deposition annealed HfO_2_/Al_2_O_3_/InSb at 400 °C for the forward and the reverse scan.

To analyze the defect state quantitatively, we investigated the hysteresis of the *C*–*V* peak and flat band voltage (*V*_fb_)^13^. The data showed that there is some difference in the quantity of fixed charges between the two samples. Figure 4 shows the change in *V*_fb_ obtained from forward and reverse sweeps; this change is attributed to the difference in trap and de-trap charging states, which can be affected by the quantities of defects. The difference in *V*_fb_ between HfO_2_/InSb and HA/InSb is ∼1.20 V, which is related to positive fixed charge, as shown in Fig. 4b and c ^14–16^. In general, the fixed charge is related to oxygen vacancies in dielectric oxide films: i.e., oxygen vacancies as a type of point defect are generated during the ALD process. Moreover, based on their bonding structure, the oxygen vacancy in Al_2_O_3_ can generate more positive fixed charge than that in HfO_2_. This means that positive fixed charge states are generated more easily in HA/InSb than in HfO_2_/InSb. Furthermore, the difference between forward and reverse *V*_fb_ shifts in HfO_2_/InSb and HA/InSb are 1.70 and 2.15 V, respectively. Since the effective electric field is affected by the fixed charge as well as the trapped charge, the trapped charge can also change *V*_fb_ of the forward and reverse sweeps^17, 18^. For this reason, if the border trap densities of HfO_2_/InSb and HA/InSb are similar, we can confirm that the larger the *V*_fb_ shift is, the harder it is for trapped charge to be de-trapped.

**Figure 5:**
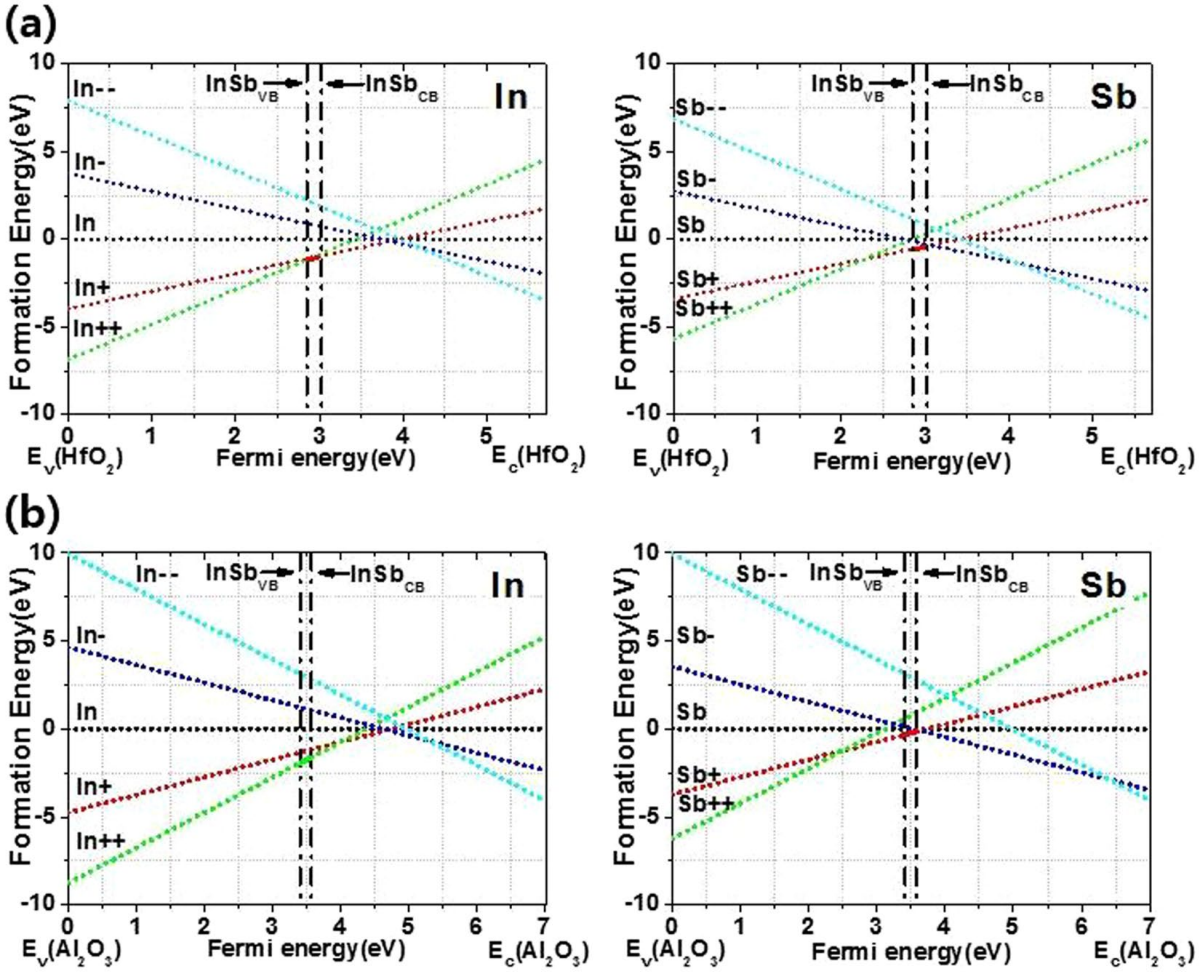
Formation energy versus Fermi level for In or Sb substituted in (**a**) the oxygen vacancy of HfO_2_ or (**b**) the oxygen vacancy of Al_2_O_3_.

DFT calculations were performed to verify the effects of In impurities in HfO_2_ and Al_2_O_3_. Figure 5a and b provide information on the formation energies for charged states of oxygen vacancy in HfO_2_ and Al_2_O_3_ films. Since the oxygen vacancy can be substituted by In or Sb, the formation energy of the oxygen vacancy substituted by In or Sb is also calculated. Briefly, Table 1 lists the formation energies of impurity states in the bandgap of InSb. According to Table 1, the In++ state formed in Al_2_O_3_ is more stable than the other states that are formed in Al_2_O_3_. The In++, In+, and VO+ states formed in HfO_2_ are more stable than the other states that are formed in HfO_2_. This means that In++ fixed charge states are easily generated in oxygen vacancies of Al_2_O_3_, while In++, In+ and VO+ charge states are easily formed in oxygen vacancies of HfO_2_. As a result, the Al_2_O_3_ passivation layer has more positive fixed charge states such as In++ than does the HfO_2_ layer, which causes the difference in *V*_fb_ between HfO_2_/InSb and HA/InSb.

**Table 1:** Formation energy at the InSb Fermi level for In or Sb substituted in the oxygen vacancy of HfO_2_ or that of Al_2_O_3_.

Formation energy in HfO_2_					
	++	+	0	−	− −
In	−1.100	−1.095	0	0.892	2.196
Sb	0.042	0.539	0	−0.117	1.138
VO	−0.558	−0.948	0	0.466	1.777
**Formation energy in Al**_2_ **O**_3_					
	++	+	0	−	− −
In	−1.675	−1.206	0	1.104	2.878
Sb	0.839	−0.194	0	0.002	2.873
VO	1.209	0.807	0	3.051	5.293

**Figure 6:**
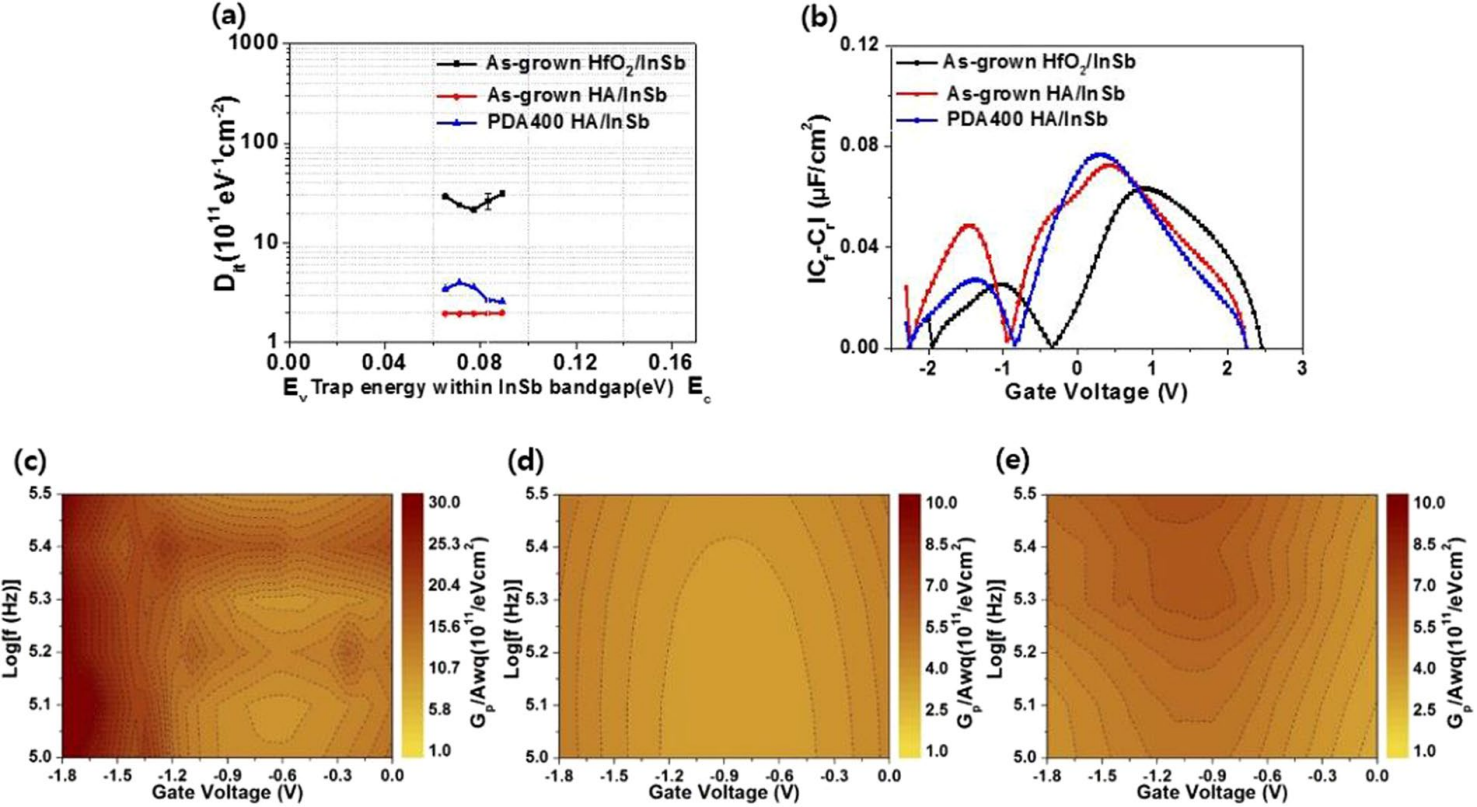
(**a**) Interface trap density (*D*_it_) from conductance results of as-grown and post-annealed HfO_2_/InSb and HA/InSb. (**b**) Effective border trap density calculated from the difference in capacitance between forward and reverse *C*–*V* sweeps at 100 kHz (|*C*_f_ − *C*_r_|, where *C*_f_ is the capacitance of the forward sweep and *C*_r_ is the capacitance of the reverse sweep) and parallel conductance (*G*_*p*_/ω*qA*) vs voltage characteristics of (**c**) as-grown HfO_2_/InSb, (**d**) as-grown HA/InSb, and (**e**) post-annealed HA/InSb at 400 °C.

To analyze the interface state between the oxide and the semiconductor, we calculated the interface trap density in each sample using the *C*–*V* curves. The interface trap density (*D*_it_) shown in Fig. 6a is associated with the quantity of oxygen vacancies located at the interface. *D*_it_ values of HfO_2_/InSb and HA/InSb were determined by using the conductance method, which is related to capacitance (*C*_m_) and conductance (*G*_m_). In the conductance method, both trapping and de-trapping of the charge carrier occur when the Fermi level of InSb is aligned with the interfacial trap states^9^. The maximum measured *D*_it_ level in HfO_2_/InSb is ∼64 × 10^11^ eV^−1^ cm^−2^. In contrast, the level is dramatically reduced in HA/InSb: i.e., the maximum *D*_it_ levels of HA/InSb before and after PDA at 400 °C are ∼1.9 × 10^11^ and ~4 × 10^11^ eV^−1^ cm^−2^, respectively. Moreover, after PDA at 400 °C in HfO_2_/InSb, we could not measure *C*_m_ and *G*_m_, because stable MOS characteristics in HfO_2_/InSb could not be maintained during the annealing process. However, in HA/InSb, since the Al_2_O_3_ layer improved the thermal stability of HA/InSb by reducing diffusion of elemental In, the maximum *D*_it_ of HA/InSb could be measured even after PDA at 400 °C. As a result, using an Al_2_O_3_ passivation layer reduces the *D*_it_ level by a factor of 10, which means that the number of oxygen vacancies located at the interface of the semiconductor and the oxide layer can be effectively reduced by Al_2_O_3_ passivation layer.

In addition to *D*_it_, the border-trap density were calculated, as shown in Fig. 6b ^19, 20^. The border-trap density in HfO_2_/InSb is similar to the density in HA/InSb before PDA at 400 °C. However, after PDA at 400 °C, the density in HfO_2_/InSb could not be measured owing to degradation of the interfacial structure of the HfO_2_/InSb sample, as previously mentioned, whereas a slightly increased border-trap density in the HA/InSb sample could be obtained because the interfacial structure can be maintained during the PDA process. Since the difference in border-trap density before and after PDA at 400 °C resulted from the increase in point defects, the change in border trap density induces an increase in leakage current related to percolation and Poole-Frenkel (P-F) tunneling. The parallel conductance contour data (*G*_p_/*A*ω*q*, where *A* is the area of the contact metal, ω is 2π*f*, and *q* is the electron charge) as functions of frequency and gate voltage are shown in Fig. 6c,d, and e. The *G*_p_/*A*ω*q* value of HfO_2_/InSb is high overall in the depletion region and its maximum value is greater than ∼19.7 × 10^11^ eV^−1^ cm^−2^ at 10^5.4^ Hz, as shown in Fig. 6a, whereas that of HA/InSb is relatively low in the region, compared with that of HfO_2_/InSb, and its maximum value is ∼4.5 × 10^11^ eV^−1^ cm^−2^ at 10^5.5^ Hz. Although, after PDA at 400 °C, the *G*_p_/*A*ω*q* value of HA/InSb is slightly increased, overall the *G*_p_/*A*ω*q* value of HA/InSb is still low, compared to that of HfO_2_/InSb. As a result, the HfO_2_/Al_2_O_3_ stack structure effectively reduces *D*_it_ and the parallel conductance as well as it very practically controls the border trap density after the annealing process up to 400 °C.

**Figure 7:**
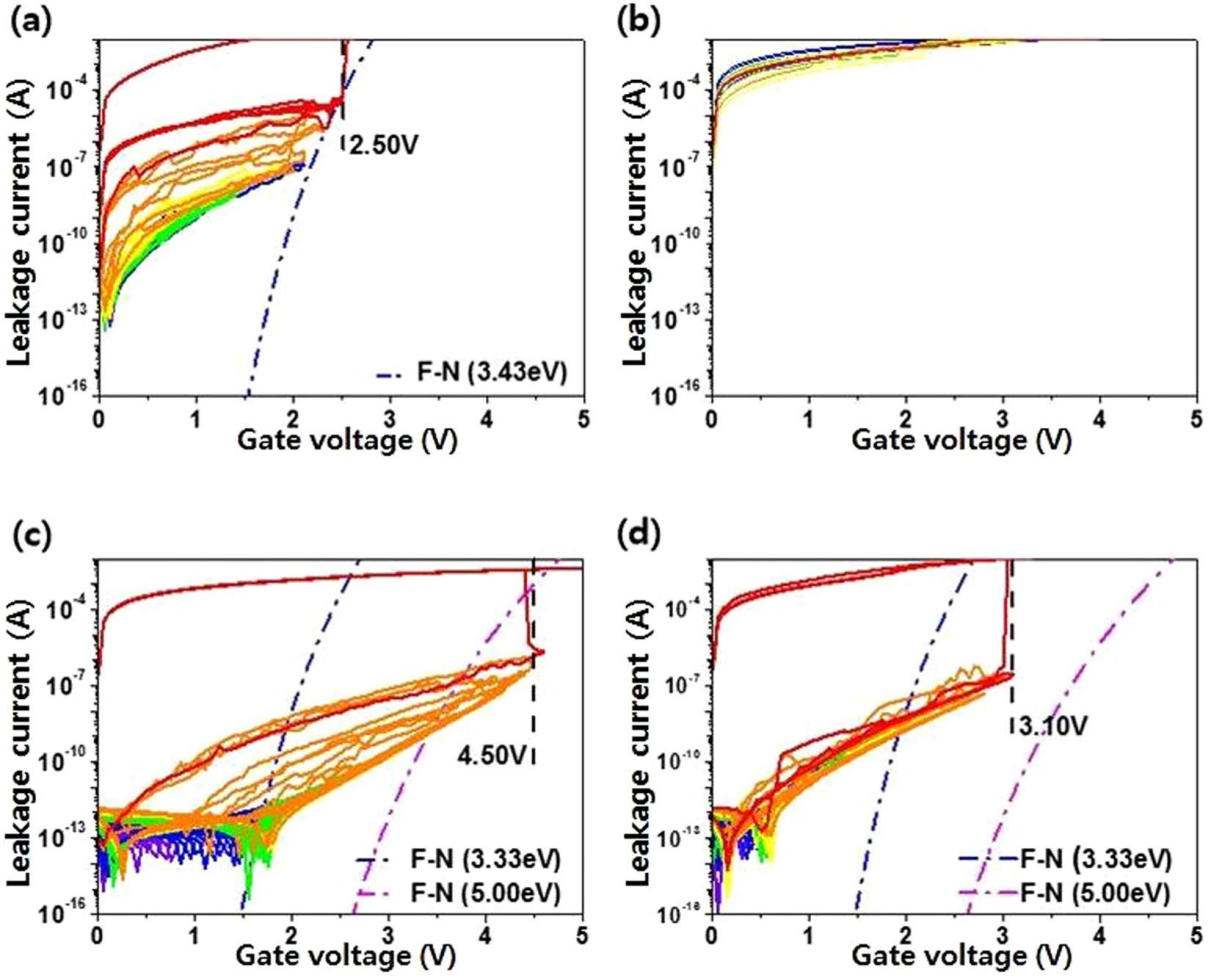
Stress-induced leakage current characteristics of (**a**) as-grown HfO_2_/InSb, (**b**) post-annealed HfO_2_/InSb at 400 °C, (**c**) as-grown HA/InSb, and (**d**) post-annealed HA/InSb at 400 °C.

To investigate the effect on the leakage current as well as the charge trapping caused by the interfacial passivation layer, we measured the stress-induced leakage current (SILC) of HfO_2_/InSb and HA/InSb, which is associated with electrical reliability under voltage stress, as shown in Fig. 7. Both forward and reverse *I*–*V* were measured as a function of ramp voltage. In HfO_2_/InSb, the reversible leakage current was maintained within a ramp voltage range from 0.5 to 2 V with a 0.05 V ramp step, as shown in Fig. 7a. After increasing the voltage above 2 V, the leakage path was consistently generated and the HfO_2_/InSb sample reached breakdown at 2.5 V. Moreover, the Fowler–Nordheim tunneling (F-N) current at a voltage above ∼2.0 V was induced. The change in F-N tunneling with the applied voltage was analyzed. F-N tunneling of an electron or hole is given by

5 $${J}_{{\rm{FN}}}=\frac{{q}^{3}}{16{\pi }^{2}\hslash {\varphi }_{{\rm{b}}}}{F}_{{\rm{ox}}}^{2}\exp \,[-\frac{4}{3}\frac{{(2{m}_{{\rm{ox}}}^{\ast })}^{\frac{1}{2}}{\varphi }_{{\rm{b}}}^{\frac{3}{2}}}{\hslash q}\frac{1}{{F}_{{\rm{ox}}}}]$$

where *q* is the electron charge, *ħ* is the reduced Planck’s constant, *m* ^*^_ox_ is the electron effective mass in the oxide layer, *ϕ*_b_ is the barrier height at the semiconductor–oxide interface, and *F*_*ox*_ is the electric field across the oxide^21^. The barrier height was obtained by using the valance band structure of XPS data and Reflective Electron Energy Loss Spectroscopy (REELS) spectra (see Supporting Fig. S2). Based on the reported values in the F-N tunneling equation, we used 0.1 ± 0.03 for *m* ^*^_ox_^22^ and 3.43 eV for *ϕ*_*b*_ in HfO_2_/InSb. After PDA at 400 °C, the sample initially broke down because the deterioration of interfacial characteristics occurred by the dissociation of InSb and the elements’ diffusion through the film. Furthermore, two tunneling effects associated with F-N tunneling as well as direct tunneling through the trap may be included in the *I*–*V* curve. Based on the reported data for SILC, the defect states can affect the leakage current in two ways. The first is trap-assisted tunneling because tunneling electrons captured by the trap states are emitted to the gate metal. The second is percolation caused by electrons hopping to sequentially lower energy trap states (multi-trap path) before emission to the gate metal by tunneling^23–25^. These two processes, which are closely related to the defect states, can induce the leakage current, which critically degrades device operation. In particular, given the gradually increased leakage current level as the applied stress increases in HfO_2_/InSb, defects can be generated continuously through the whole oxide in HfO_2_/InSb. Therefore, this characteristic SILC indicates the increase in leakage current through the multi-trap path: The percolation process more dominantly affects the increased leakage current, compared with the trap-assisted tunneling process. However, in HA/InSb, a reversible leakage current was maintained within a ramp voltage range from 0.5 to 4 V with a 0.05 V ramp step, as shown in Fig. 7c. Unlike HfO_2_/InSb, when an electrical stress >4 V was applied, an F-N tunneling current in HA/InSb was not observed in the enhanced current region: The SILC of HA/InSb could not be fitted using the *J*_FN_ equation. In addition, an F-N tunneling current in HA/InSb was not observed after PDA at 400 °C. When an electrical stress >4.0 V was applied, the leakage current followed different curve shapes, compared with the case of F-N tunneling in Fig. 7a. The different curve shapes for the SILC are related to the defect states caused by electrical stress: i.e., quantum mechanical tunneling and trap-assisted tunneling can occur through the generated defects. Since the tunneling processes are more related to the bulk defects, not to the interfacial defects, the increase in border trap density well supports the change in the SILC curve. Another interesting finding is that, after PDA at 400 °C, the reversible leakage current was maintained up to the applied stress voltage of 3.1 V, as shown in Fig. 7d: i.e., no degraded characteristics of current shape up to the applied stress voltage of ∼3.1 eV are observed. Comparing the leakage current before PDA at 400 °C to that after PDA at 400 °C, we see that the line shapes of the leakage current levels over the applied stress voltage of ∼4.3 eV in Fig. 7c are similar to those in Fig. 7d. Therefore, the results indicate that the cause for the leakage path after the electrical stress before PDA is similar to that after PDA in HA/InSb. As a result, in addition to the advantage of the larger barrier height of Al_2_O_3_ compared to that of HfO_2_, the effective reduction of the leakage current level using an Al_2_O_3_ passivation layer is mainly caused by the control of the interfacial reaction and elemental diffusion. Finally, we can effectively improve the thermal stability of a MOS capacitor by using an Al_2_O_3_ passivation layer.

## Conclusions

In summary, we investigated the electrical properties and thermal stability in the stack structure HfO_2_/Al_2_O_3_/InSb by atomic layer deposition. We obtained more detailed analysis data related to diffusion and chemical reaction analysis of the HfO_2_/InSb system than previously reported papers. Moreover, based on these in-depth analysis, we could provide new information on the interface states that induce the charge trapping. An interfacial reaction is generated during the ALD process for HfO_2_ grown on InSb, whereas the reaction is significantly reduced by using an Al_2_O_3_ passivation layer on InSb even during PDA. Unfortunately, we could not obtain reliable data in the single-layer HfO_2_ after PDA at 400 °C. The results clearly suggest that significant deterioration of the film quality occurred during PDA in the single-layer HfO_2_, but not in the stack structure of the HA/InSb sample. Since the Al_2_O_3_ layer prevents elemental In from diffusing out through the HfO_2_/Al_2_O_3_ stack structure, the dielectric characteristics of HA/InSb are stably maintained even after PDA at 400°. Although the Al_2_O_3_ passivation layer gave rise to positive fixed charge, which negatively shifts *V*_fb_, *D*_it_ can be reduced by dramatically decreased diffusion of elemental In. More specifically, the amount of elemental In on the HA/InSb surface is significantly reduced by 80% on the HfO_2_/InSb surface, and the value of *D*_it_ of HA/InSb is also clearly lower by a factor of 10 compared to that of HfO_2_/InSb. Finally, although the electrical properties based on InSb are not as good as those based on the other III-V materials, the Al_2_O_3_ passivation layer effectively reduces the leakage current and dramatically increases the MOS capacitor performance and thermal stability in the InSb system. The results herein suggest that the defect states generated by diffusion are fatal for operation of a MOS capacitor owing to the narrow bandgap of InSb, 0.17 eV. In the case of a narrow bandgap, the operation of a MOS device can be more severely affected by the defect states within the bandgap, compared with the other III–V compound semiconductor materials that have wide bandgaps of >0.5 eV^25–29^. Therefore, it is important to control the diffusion of elemental In, which generates the defect states in the InSb system. Based on these results, to achieve better device performance and thermal stability for MOSFETs, we conclude that HfO_2_–Al_2_O_3_ stacked structures can be a promising suggestion for MOS structures using InSb with a narrow bandgap, because the defect generation within the narrow bandgap severely affects the electrical properties.

## Electronic supplementary material

Supporting information
