# Supplementary material for: Electrical properties and thermal stability in stack structure of HfO2/Al2O3/InSb by atomic layer deposition
Source: Sci Rep. 2017 Sep 12;7:11337. doi: 10.1038/s41598-017-09623-1 (PMC5595993; doi:10.1038/s41598-017-09623-1)
Supplement: Supplementary file 1 — Supporting information [file 41598_2017_9623_MOESM1_ESM.doc]

**Supporting Information**

**Electrical properties and thermal stability in stack structure of HfO_2_ /Al_2_O_3_ /InSb­**

**by atomic layer deposition**

Min Baik^a^, Hang-Kyu Kang^a,c^, Yu-Seon Kang^a^, Kwang-Sik Jeong^a^, Youngseo An^b^, Seongheum Choi^b^,

Hyoungsub Kim^b^, Jin-Dong Song^c^, and Mann-Ho Cho^a*^

^a^Institute of Physics and Applied Physics, Yonsei University, Seoul, 120-749, Republic of Korea

^b^School of Advanced Materials Science and Engineering, Sungkyunkwan University, Suwon, 440-746, Republic of Korea

^c^Center of Opto-electronic Materials, Korea Institute of Science and Technology, Seoul, 02792, Republic of Korea

*E-mail : mh.cho@yonsei.ac.kr

Table S1. Top: Raw data of TOF SIMS(time of flight secondary ion mass spectrometry)

Bottom: The ratio of ion quantity of TOF SIMS


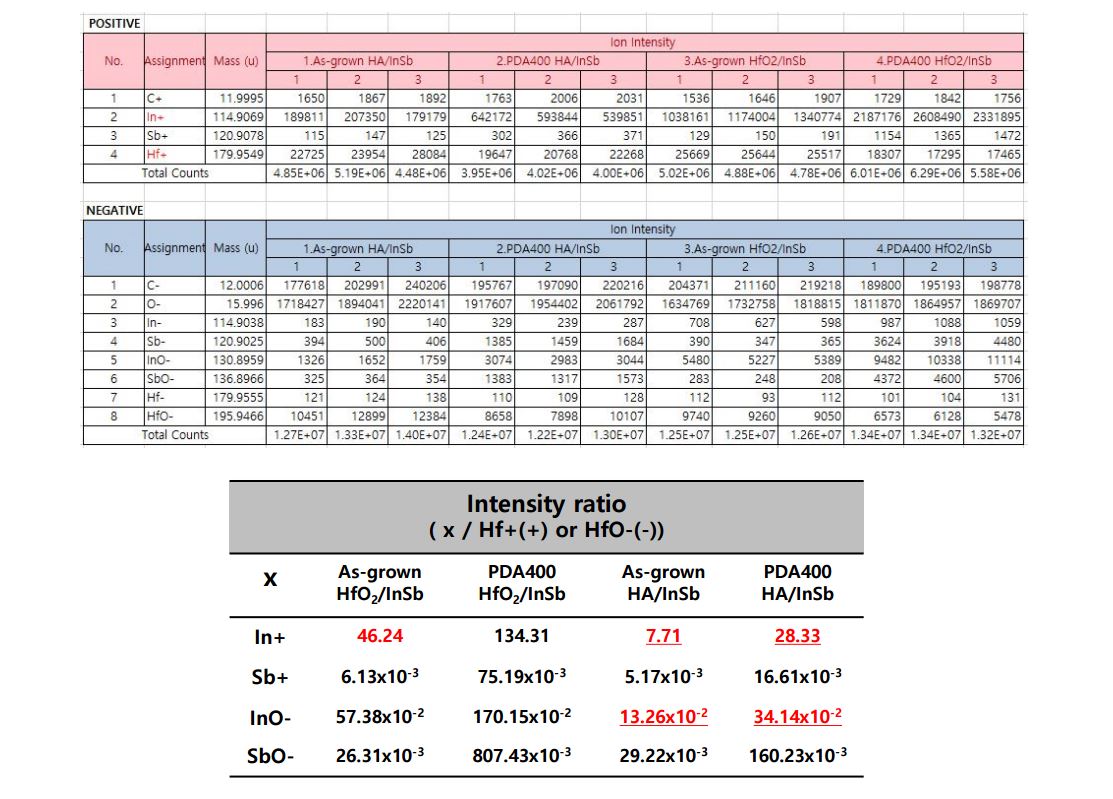


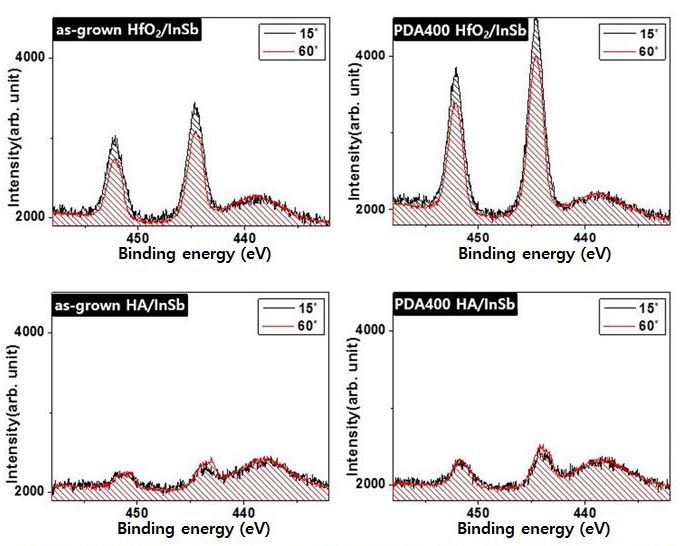


Figure S1. XPS In 3d core-level spectra with two takeoff angles(15° and 60°)


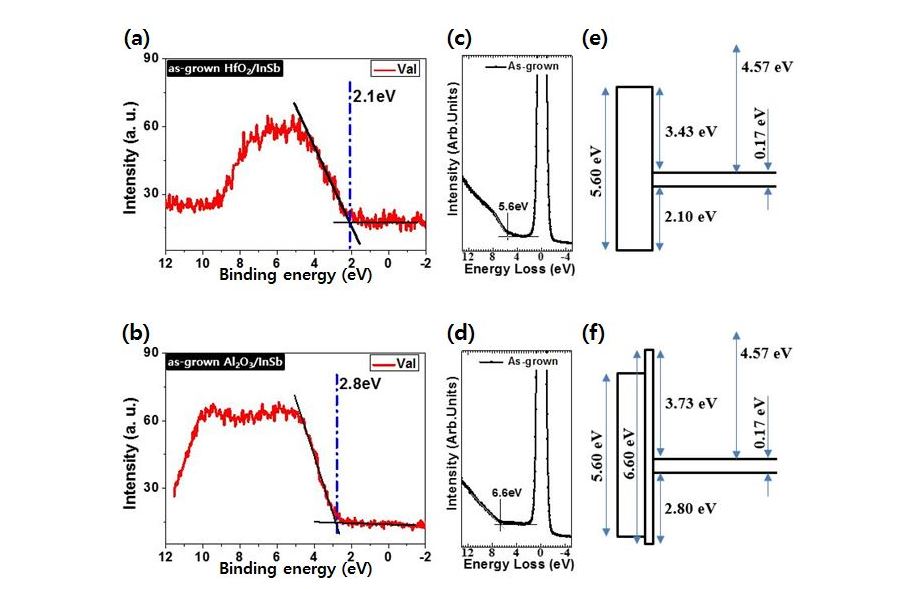


Figure S2. Left side: XPS data for (a) 5nm thick HfO_2_ and (b) 5nm thick Al­_2_O_3_. Middle side: REELS

spectra for (c) 5nm thick HfO_2_ and (d) 5nm thick Al_2_O_3_. Right side: band alignment for (e) 5nm thick

HfO_2_/InSb and (f) 5nm thick HA/InSb
